# Lipid Profile, Obesity Indicators and Cardiometabolic Risk Factors in School-Aged Children and Adolescents: Sex-Specific Associations

**DOI:** 10.3390/jcm14186677

**Published:** 2025-09-22

**Authors:** Rafał Baran, Joanna Baran, Justyna Leszczak, Anna Bartosiewicz, Justyna Wyszyńska

**Affiliations:** Faculty of Health Sciences and Psychology, Collegium Medicum, University of Rzeszów, Kopisto Avenue 2a, 35-959 Rzeszów, Poland; jbaran@ur.edu.pl (J.B.); jleszczak@ur.edu.pl (J.L.); abartosiewicz@ur.edu.pl (A.B.)

**Keywords:** adolescents, cardiometabolic risk factors, children, hypertension, obesity

## Abstract

**Background**: Childhood obesity and cardiometabolic disturbances are growing global health concerns. This study aimed to assess the prevalence of excess body weight, body fat, and selected cardiometabolic risk factors in school-aged children and adolescents, focusing on sex- and age-related differences. **Methods**: A cross-sectional study was conducted among 318 Polish participants aged 6–17 years, including 169 children (6–12 years) and 149 adolescents (13–17 years). Anthropometric, blood pressure (BP), and fasting blood lipid and glucose measurements were collected and analyzed by age group (children 6–12 years; adolescents 13–17 years) and sex. **Results**: The prevalence of overweight and obesity was 18.5% (BMI-based) and 26.1% (body fat-based). Abdominal obesity and stage I–II hypertension were observed in 24.5% and 23.6% of participants, respectively. Children had higher rates of excess body fat, abdominal obesity, elevated BP, and lipid abnormalities than adolescents. Among adolescents, girls more frequently presented with borderline/high total cholesterol and Low-Density Lipoprotein (LDL cholesterol) and borderline/low High-Density Lipoprotein (HDL cholesterol), while boys more often had elevated BP. In girls, elevated triglycerides (TGs) were independently associated with abdominal obesity (odds ratio (OR) = 2.36, *p* = 0.015) and hypertension (OR = 2.47, *p* = 0.023); no such associations were observed in boys. **Conclusions**: Cardiometabolic risk factors may appear early in life and differ by age and sex. Routine screening and early interventions, particularly targeting lipid abnormalities in girls, are essential to prevent long-term health consequences.

## 1. Introduction

Childhood obesity and the associated cluster of cardiometabolic disturbances represent one of the most pressing public health challenges of the 21st century. This issue is global in scope and affects countries with diverse socioeconomic profiles, particularly in urban areas. The substantial increase in the prevalence of excess body weight among pediatric populations worldwide contributes to a greater risk of cardiovascular disease later in life [1]. The World Health Organization (WHO) data indicates that, in 2024, 35 million children under the age of 5 were overweight [2]. Children with overweight and obesity are more likely to remain obese into adulthood and are at greater risk of developing non-communicable diseases such as diabetes and cardiovascular disease at a younger age [3].

Childhood obesity has a significant impact on both physical and mental health. It can lead to serious health conditions, including type 2 diabetes, cardiovascular problems, asthma, obstructive sleep apnea, hypertension, non-alcoholic fatty liver disease, gastroesophageal reflux disease, and various psychosocial difficulties. Preventive and therapeutic interventions targeting childhood obesity are essential for reducing the burden of associated comorbidities [4].

Global trend analyses indicate that over the past four decades, the number of children and adolescents with obesity has increased more than tenfold—an unprecedented phenomenon in the history of medicine [5]. In Poland, trends in the prevalence of overweight and obesity among eight-year-old children were analyzed for the period 2016–2022/2023. A linear increase in the proportion of children with excess body weight was observed between 2016 and 2021, followed by a decrease in 2022/2023; however, the values remained higher than those recorded before the COVID-19 pandemic [6]. According to scientific reports, the percentage of children with overweight and obesity in Poland ranged from 16.4 to 26.4, with the highest percentage recorded among teenagers aged 10–13 [7,8,9].

This growing epidemic has profound clinical implications. Robust scientific evidence from long-term cohort studies such as the Bogalusa Heart Study shows that atherosclerotic processes begin in childhood [10]. Risk factors such as dyslipidemia, hypertension, and insulin resistance emerging early in life tend to persist into adulthood—a phenomenon known as tracking—and form the foundation for the development of atherosclerotic cardiovascular disease and its clinical manifestations, including myocardial infarction and stroke [11].

Despite increasing awareness, few Polish studies have comprehensively examined the associations between detailed obesity indicators and lipid profiles in pediatric populations using multivariate modeling. Existing analyses have often been limited to single measures such as body mass index (BMI), rarely incorporating more precise data on body composition obtained through bioelectrical impedance analysis (BIA), abdominal obesity indices (which are particularly relevant for metabolic risk), and BP values. Moreover, few studies have simultaneously applied a cross-sectional approach and multivariate regression models with separate analyses for girls and boys. This is a critical gap, as sex-specific differences in hormonal regulation—especially during puberty—can differentially influence lipid profiles, fat distribution, and other cardiometabolic risk factors [12,13]. In 2022, the prevalence of registered hypertension among children in Poland was 0.4%.

This study aims to address this research gap by providing a novel and comprehensive analysis based on objective anthropometric, clinical, and biochemical measurements in a representative sample of Polish children and adolescents. The uniqueness of the study lies in the integration of multiple obesity indicators and the in-depth, sex-specific evaluation of their associations with lipid profiles.

Accordingly, the objective of this cross-sectional study was to assess the prevalence of obesity and cardiometabolic risk factors among school-aged children and adolescents and to explore sex-specific associations between lipid profile abnormalities and selected anthropometric and clinical parameters.

## 2. Materials and Methods

### 2.1. Study Participants

The study employed a cross-sectional design and was conducted in 2024 among children and adolescents from the Podkarpackie region in southeastern Poland. Participants were recruited from both primary and secondary schools using a stratified random sampling technique to reflect the demographic distribution of rural and urban settings. In total, 12 schools (7 primary and 5 secondary) from both rural and urban areas of the Podkarpackie region were invited to participate. This ensured proportional representation of the regional demographic structure. Before any procedures were initiated, written informed consent was obtained from both the students and their parents or legal guardians. The study protocol received approval from the Bioethics Committee of University of Rzeszow (Resolution No. 2023/12/062W, dated 6 December 2023). All procedures were carried out in line with the ethical standards of the Declaration of Helsinki.

**Figure 1:**
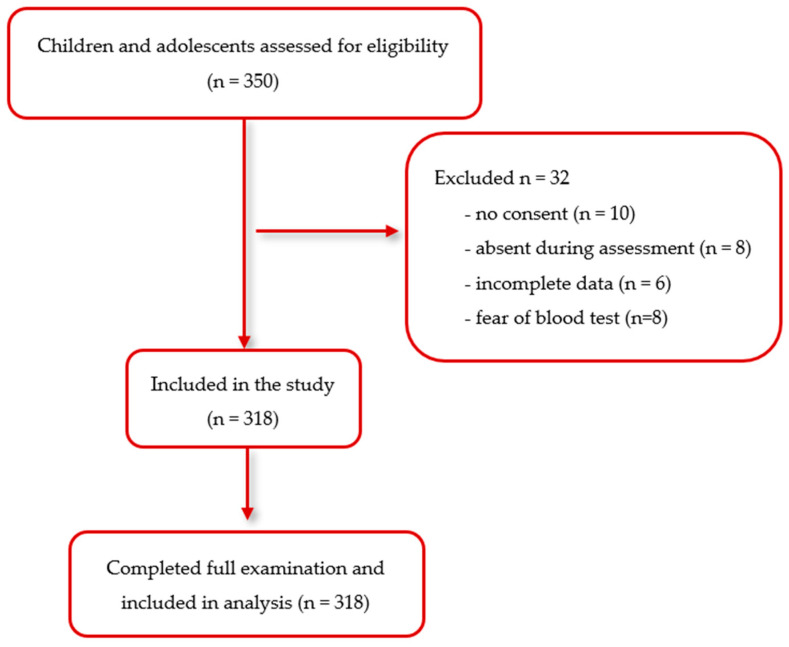
Flow diagram of participant screening, inclusion, and completion of the examinations.

The required sample size was estimated in advance using statistical methods, indicating that at least 288 participants would be necessary to ensure reliable estimates with a 95% confidence level and a 5% margin of error. To account for possible dropouts, incomplete assessments, or difficulties during examinations, the target sample was increased to 350 children and adolescents. After applying the exclusion criteria and removing cases with incomplete data or participant withdrawal, the final analytical sample consisted of 318 individuals, providing adequate statistical power and representativeness. For the purpose of analysis, participants were divided into two age groups: children (6–12 years) and adolescents (13–17 years). These age categories align with common age strata used in international epidemiological studies of pediatric obesity and cardiometabolic risk, such as age groupings starting from 6 years for children and up to 18 for adolescents [doi: 10.1210/clinem/dgz195; doi: 10.1001/jamapediatrics.2024.1576]. The recruitment process, inclusion flow, and reasons for exclusion are detailed in Figure 1 (Flow diagram of participant screening, inclusion, and completion of the examinations).

Participants were excluded from the study if they presented with medical conditions contraindicating participation in BIA (such as implanted metal devices or pacemakers) or blood collection procedures (including coagulation disorders or a severe fear of needles). Additionally, individuals with any acute illness or chronic disease known to influence metabolic or anthropometric outcomes (e.g., diabetes, endocrine disorders, or genetic syndromes associated with obesity) were not eligible for inclusion. The final sample consisted exclusively of healthy children and adolescents, thus ensuring that the results reflected typical physiological variation rather than disease-related alterations.

A structured questionnaire administered to parents or legal guardians was used to assess participants’ health status, including the presence of chronic conditions and the use of medications such as antihypertensive or lipid-lowering agents. Based on the collected data, no participants were reported to be receiving such treatment.

### 2.2. Measurements

#### 2.2.1. Blood Profile

Capillary blood samples were obtained in the morning hours (between 7:00 and 10:00 a.m.) following an overnight fast of at least 10 h. The collection was performed via finger prick by a qualified nurse using standardized procedures to ensure accuracy and participant comfort. Immediate, on-site analysis was conducted using the CardioChek^®^ PA Analyzer (PTS Diagnostics, Whitestown, IN, USA), a point-of-care device validated for field use in the assessment of lipid profiles and glucose concentrations. The CardioChek^®^ PA Analyzer provides quantitative measurements of total cholesterol, TG, HDL cholesterol level, and fasting glucose concentration. LDL cholesterol level was calculated using the Friedewald formula. The device has demonstrated good agreement with standard laboratory-based biochemical assays [14]. This dry chemistry analyzer has been recommended by WHO in the STEPS Surveillance Manual as a reliable tool for large-scale population screening of blood glucose, total cholesterol, TG, and HDL cholesterol [15,16].

Lipid profile results were classified according to pediatric reference thresholds, distinguishing acceptable and borderline/high values. For total cholesterol, values < 170 mg/dL were considered acceptable, 170–199 mg/dL as borderline, and ≥200 mg/dL as high. For LDL cholesterol, the thresholds were <110 mg/dL (acceptable), 110–129 mg/dL (borderline), and ≥130 mg/dL (high). For HDL cholesterol, values > 45 mg/dL were considered acceptable, 35–45 mg/dL as borderline, and <35 mg/dL as low. For triglycerides (TG), age-dependent thresholds were applied: for children aged 0–9 years, values were classified as acceptable (<75 mg/dL), borderline (75–99 mg/dL), and high (≥100 mg/dL), while for adolescents aged 10–19 years, the corresponding values were <90 mg/dL, 90–129 mg/dL, and ≥130 mg/dL [17]. Fasting glucose concentration was evaluated based on established pediatric criteria, defining values of 70–99 mg/dL as normal, <70 mg/dL as below the norm, and >99 mg/dL as elevated glycemia [18].

#### 2.2.2. Blood Pressure

Resting BP was assessed using a validated automated oscillometric monitor (Welch Allyn, Skaneateles Falls, NY, USA), following the recommendations of the European Society of Hypertension for pediatric populations [19]. Prior to measurement, each participant remained seated in a relaxed, upright position for a minimum of five minutes to ensure resting conditions. BP was measured on the right arm, supported at heart level, using an appropriately sized cuff selected according to individual mid-arm circumference. Three consecutive readings of systolic blood pressure (SBP) and diastolic blood pressure (DBP) were obtained at 1 min intervals in a quiet setting. The average of the last two readings was used for subsequent analyses to minimize potential measurement variability.

BP classification was based on percentile grids specific to age, sex, and height, in accordance with the centile reference charts developed in the Polish OLAF study (a nationwide growth reference study) [20].

#### 2.2.3. Anthropometric Measurements and Body Composition

Anthropometric measurements included body height, body weight, waist circumference (WC), and hip circumference (HC). Height was measured to the nearest 0.1 cm using a portable stadiometer (Tanita HR-001, Tokyo, Japan), with participants standing barefoot in a standardized upright posture. WC was measured midway between the lower margin of the last palpable rib and the top of the iliac crest, while HC was assessed at the widest point over the buttocks, using a flexible, non-stretchable tape. All circumferences were recorded to the nearest 0.1 cm. Measurements were performed according to the International Society for the Advancement of Kinanthropometry (ISAK) protocol [21].

The waist-to-hip ratio (WHR) was calculated to assess central fat distribution. Abdominal obesity was defined as WC ≥ 90th percentile for age and sex, based on Polish reference values from the OLAF/OLA project [22]. HC was analyzed descriptively, as no diagnostic thresholds were applied in this study.

Body weight was assessed with a segmental body composition analyzer (Tanita MC-780 P MA; Tanita Corporation, Tokyo, Japan), with 0.01 kg accuracy. BMI was calculated using the standard formula (weight in kilograms divided by height in meters squared). BMI-for-age percentiles were derived using Polish reference values (OLAF/OLA project) [23] and used to classify weight status as follows: underweight (BMI < 5th percentile), normal weight (5th < 85th percentile), overweight (85th–<95th percentile), and obesity (≥95th percentile), in accordance with Centers for Disease Control and Prevention (CDC) growth charts [24].

#### 2.2.4. Body Composition

Body composition was evaluated via BIA using the Tanita MC-780 P MA analyzer. Measurements were conducted in the morning (between 7:00 and 10:00 a.m.), in a fasted state, with participants barefoot and wearing light indoor clothing to ensure standardized conditions. The device estimated body fat percentage, which was used to classify body fat status as follows: underfat (<2nd percentile), normal (2nd–84th percentile), overfat (85th–94th percentile), and obesity (≥95th percentile), based on sex- and age-specific reference values proposed by McCarthy et al. [25].

#### 2.2.5. Data Analysis

Statistical analyses were performed using Statistica software version 13.3 (TIBCO Software Inc., Palo Alto, CA, USA). Quantitative variables are presented as arithmetic means with standard deviations (±SD) or as medians with interquartile ranges (Me; Q1–Q3), depending on the data distribution. Qualitative variables are expressed as counts and percentages (%).

The normality of data distribution was assessed using the Shapiro–Wilk test, while the homogeneity of variances was evaluated with Levene’s test. To compare quantitative characteristics between girls and boys, the independent samples Student’s *t*-test was used when the assumptions of normality and homogeneity of variances were met. If the assumption of equal variances was violated, Welch’s *t*-test was applied. For variables that did not follow a normal distribution, the non-parametric Mann–Whitney U test was used.

Associations between categorical variables were analyzed using Pearson’s chi-square test. When the expected cell count in at least one cell of the contingency table was less than 10, the Yates-corrected chi-square test was employed. For statistically significant results, the strength of association was assessed using the contingency coefficient C.

To compare mean ranks of lipid concentrations across categories of body weight status, abdominal obesity, and BP classification, the Mann–Whitney U test (for two groups) and the Kruskal–Wallis test (for three groups) were used.

Associations between lipid profile abnormalities and selected clinical parameters were examined using univariate logistic regression. These analyses were performed separately for each lipid variable (TG, LDL, HDL) as independent variables, and ORs with 95% confidence intervals (CIs) were calculated for the occurrence of abdominal obesity, abnormal glucose levels, and hypertension. Analyses were conducted separately for girls and boys.

Additionally, multivariate logistic regression models were constructed to assess the simultaneous effects of TG, LDL cholesterol, and HDL cholesterol classifications on the presence of each condition (abdominal obesity, hypertension, abnormal glucose levels).

A *p*-value of <0.05 was considered statistically significant for all analyses.

## 3. Results

Table 1 presents anthropometric, clinical, and biochemical characteristics of the study population stratified by age group (6–12 years and 13–17 years) and sex. Among younger children, no significant sex differences were observed across any measured parameters. In contrast, among adolescents (13–17 years), boys had significantly higher height, weight, WC and HC, WHR, and SBP compared to girls (all *p* < 0.01). Girls in this age group had significantly higher total and HDL cholesterol levels (*p* = 0.003 and *p* < 0.001, respectively). No significant sex differences were noted in fasting glucose, DBP, or TG in either age group.

Table 2 summarizes the distribution of body weight status, body fat status, and selected cardiometabolic risk factors among children, adolescents, and the total sample, stratified by sex.

The overall prevalence of overweight and obesity (based on CDC BMI percentiles) in the total sample (n = 318) was 18.5% (overweight: 10.7%, obesity: 7.8%), while based on body fat status, the combined prevalence of overfat and obesity was 26.1% (overfat: 14.8%, obesity: 11.3%). Abdominal obesity was present in 24.5% of participants, and stage I–II hypertension was found in 23.6% of the total group. Abnormal fasting glucose levels (either low or high) were observed in 32.7%, and lipid abnormalities were also common: borderline/high TG in 44.0%, borderline/low HDL cholesterol in 36.2%, borderline/high LDL cholesterol in 22.0%, and borderline/high total cholesterol in 16.7%.

Children aged 6–12 years demonstrated a higher prevalence of several cardiometabolic abnormalities compared to adolescents. Higher proportions of overfat status, abdominal obesity, stage I–II hypertension, abnormal TG, LDL cholesterol, and total cholesterol were observed in the younger group. In contrast, adolescents had a higher prevalence of abnormal fasting glucose and borderline/low HDL cholesterol levels. These findings indicate that cardiometabolic risk factors may already be present at an early age and should be monitored from childhood.

Among children aged 6–12 years, no statistically significant sex differences were observed for any variable, including body weight and body fat status, BP, glucose, or lipid categories (*p* > 0.05 for all comparisons).

In adolescents aged 13–17 years, girls had a significantly higher prevalence of borderline/low HDL cholesterol (*p* = 0.002), borderline/high LDL cholesterol (*p* = 0.005), and borderline/high total cholesterol (*p* = 0.040) compared to boys. No significant sex differences were found in the distribution of weight or fat categories, BP status, glucose levels, or TG.

Boys also had a higher prevalence of stage I–II hypertension compared to girls (15.7% vs. 7.6%, *p* = 0.002). No significant sex differences were found in the distribution of weight or fat categories, glucose levels, or TG.

Table 3 presents mean values and standard deviations for total cholesterol, HDL cholesterol, LDL cholesterol, and TG according to selected anthropometric and clinical classifications in the total study sample.

A significantly lower HDL cholesterol level was observed in participants with overfat or obesity compared to those with underfat or normal body fat (*p* = 0.045). Additionally, participants with abdominal obesity had significantly higher TG levels (*p* = 0.025). No statistically significant differences were found in total cholesterol or LDL cholesterol across any classification, and no differences in lipid parameters were observed between BP categories.

Table 4 presents the results of univariate logistic regression analyses assessing the association between abnormal lipid levels and selected health outcomes in girls.

Girls with borderline/high TG levels had significantly higher odds of abdominal obesity (OR = 2.09, *p* = 0.033) and stage I–II hypertension (OR = 2.18, *p* = 0.045) compared to those with acceptable levels. No statistically significant associations were observed for LDL or HDL cholesterol levels with any of the outcomes. However, non-significant trends were noted toward higher rates of abdominal obesity in girls with borderline/low HDL cholesterol and toward elevated glucose in those with borderline/high TG. No statistically significant associations were found between any lipid abnormalities and the analyzed health outcomes in boys (*p* > 0.05 for all comparisons).

Table 5 presents the results of multivariate logistic regression models examining the associations between lipid profile abnormalities (TG, LDL cholesterol, and HDL cholesterol) and selected health outcomes in girls, including abdominal obesity, elevated fasting glucose, and stage I–II hypertension.

Among the three lipid parameters included in each model, abnormal TG levels were significantly associated with abdominal obesity (*p* = 0.015) and stage I–II hypertension (*p* = 0.023) in girls. These associations remained significant after adjusting for LDL and HDL cholesterol levels.

None of the associations between lipid profile abnormalities and the analyzed health outcomes reached statistical significance in boys (*p* > 0.05 for all comparisons).

## 4. Discussion

This study revealed a high prevalence of lipid and cardiometabolic abnormalities in the examined pediatric population, particularly among children aged 6–12 years. The TG levels were significantly associated with abdominal obesity and hypertension in girls, but not in boys, highlighting the presence of sex-specific differences in cardiovascular risk factors already during developmental age. Although sex differences in obesity-related cardiometabolic risk have been described in other populations, data from Central and Eastern Europe remain limited. Our study contributes to the literature by integrating several obesity indicators (BMI, BIA, abdominal obesity) with lipid parameters in a Polish cohort, thus enhancing the understanding of sex-specific risk profiles in this region.

The results showed that excess body weight, based on BMI percentile charts, affected 18.5% of participants (10.7% overweight and 7.8% obesity). These values are consistent with other Polish reports, such as those from the Institute of Mother and Child within the WHO European Childhood Obesity Surveillance Initiative (COSI), which also highlight the alarming scale of the problem [6]. Globally, studies indicate a 1.5-fold increase in obesity prevalence among children and adolescents between 2012 and 2023 compared to 2000–2011, with current rates reaching 8.5% (95% CI: 8.2–8.8). Notable differences in obesity prevalence were observed after stratification by 11 risk factors, with children and adolescents with obesity exhibiting higher risks of depression and hypertension. Pooled estimates for overweight and excess body weight in youth were 14.8% (95% CI: 14.5–15.1) and 22.2% (95% CI: 21.6–22.8), respectively [26].

Importantly, body composition assessment via BIA identified a higher percentage of adolescents with excess fat mass (26.1% in total), supporting the notion that BMI may underestimate the true metabolic risk associated with obesity. Similar conclusions were drawn by Farbo and Rhea, who found that BIA was a more sensitive tool for detecting adiposity in children compared to BMI [27]. Studies by Salman et al. also emphasized the need for caution when diagnosing obesity based solely on BMI, as the proportion of fat mass, not just weight, plays a crucial role in metabolic health. Their findings showed discordance between BMI and body fat percentage measured via BIA [28].

In this study, the prevalence of specific risk factors was high: hypertension was observed in 23.6% of participants, abdominal obesity in 24.5%, and borderline/high TG levels in 44.0%. Hypertriglyceridemia is more commonly observed among children with overweight or obesity compared to the general pediatric population (13.8–31.8% vs. 5.9–8.6%) and is associated with other cardiometabolic risk factors [29]. While the overall prevalence of hypertension in U.S. children is 3–5%, the rate is much higher in children with obesity, reaching up to 25% [30]. Among Ukrainian schoolchildren, elevated BP was found in 50% of those with overweight or obesity, hypertension in 13.3% of children, with prehypertension and stage 1 hypertension being twice as frequent in boys than in girls [31]. In Turkey, hypertension was diagnosed in 14.8% of adolescents aged 14–19 years, with 41.6% of those cases occurring in individuals with overweight or obesity—rates significantly associated with BMI and WC [32,33].

In Spain, over the past two decades, the proportion of children at increased cardiometabolic risk based on abdominal obesity rose from 16% to 22.6% between 1999 and 2020 [34]. This finding underscores the importance of including WC measurements in routine pediatric practice. In Greece, the prevalence of hypertension among children with general and central obesity is among the highest in Europe, reaching 49.7% [35]. These data point to a strong correlation between abdominal obesity and early cardiometabolic risk.

When compared to international data, our findings suggest that components of the metabolic syndrome—hypertension, abdominal obesity, and hypertriglyceridemia—are already intensifying during adolescence and may accumulate into adulthood. The observed high rate of hypertriglyceridemia (44%) among children and adolescents is characteristic of pediatric populations with overweight/obesity and aligns with trends reported in the U.S. and Asia. Importantly, interventions targeting children and adolescents offer a critical opportunity to halt the progression of these risk factors and mitigate their long-term impact on cardiovascular disease.

A surprising finding in our study was the higher prevalence of certain abnormalities—BIA-defined obesity, abdominal obesity, stage I and II hypertension, and borderline/high TG levels—in the younger age group (6–12 years) compared to adolescents. This may suggest that key pathophysiological processes are initiated as early as primary school age. Our observations are consistent with Spanish and Chinese studies showing significantly elevated BP and hypertension risk in overweight or obese children during early school years [36,37]. Longitudinal research on children aged 4–6 has demonstrated that those with general or central obesity show a marked increase in BP within a few years, with a 2.5–3-fold higher risk of hypertension by age 6 [38]. Furthermore, anthropometric studies have indicated that BMI and Waist-to-Height Ratio correlate with BP and TG levels in children as strongly as they do in adolescents. In some cases, these correlations are even stronger in younger children [39,40]. Longitudinal evidence confirms that early onset of obesity and lipid disturbances contributes to future cardiovascular disease, emphasizing the importance of early intervention during the early school years [41].

Our study also highlighted notable sex differences, particularly among adolescents. Boys aged 13–17 had higher SBP and a greater prevalence of hypertension. In contrast, girls in this age group had significantly higher levels of total and HDL cholesterol and were more frequently diagnosed with dyslipidemia involving HDL, LDL, and total cholesterol. These findings align with existing literature indicating a complex influence of sex hormones during puberty on lipid profiles and BP. Increases in testosterone in boys may contribute to higher BP, while estrogens in girls may modulate lipoprotein metabolism [42,43]. As McGill et al. noted, such changes are a physiological part of puberty but may lead to a persistent adverse risk profile when combined with excess body weight [44].

The most important finding of this study is the strong and independent association between elevated TG levels and both abdominal obesity (OR = 2.36) and hypertension (OR = 2.47) in girls. No such associations were found in boys. The TG level is a recognized marker of insulin resistance and lipotoxicity, with elevated levels reflecting an excess of metabolically active visceral adipose tissue [45]. These results suggest that in peripubertal girls, TG metabolism may serve as a particularly sensitive indicator of metabolic dysfunction. Research by Mauriège et al. showed that in peripubertal females, fat distribution—particularly visceral fat—was strongly associated with alterations in lipid profiles, including elevated TG and LDL-C and decreased HDL-C. These findings indicate that TG metabolism in maturing girls may be a key marker of metabolic dysfunction [46]. The lack of significant associations in boys in our study may reflect divergent hormonal pathways, different rates of maturation, or behavioral differences—all of which warrant further investigation.

Our findings suggest that TG levels may serve as a simple, cost-effective screening tool to identify girls at increased cardiometabolic risk, which could be particularly useful in primary care and school-based health programs. Early detection of such abnormalities may guide tailored interventions, including nutritional counseling and promotion of physical activity, before more severe metabolic complications develop.

Among the strengths of this study is its comprehensive design, integrating multiple obesity indicators—from standard BMI to BIA-based body composition measurements and markers of abdominal obesity. The use of objective, validated measurement tools and stratification by sex and age further strengthens the analysis.

Nonetheless, this study has certain limitations. Its cross-sectional design precludes causal inference. Another limitation is the absence of lifestyle data, such as dietary intake, physical activity, or sedentary behavior, which are known to influence cardiometabolic profiles. Including these variables in future research would allow a more comprehensive analysis of risk factors. Furthermore, lipid profiles were assessed using a portable analyzer on capillary blood samples. Although this method is validated for screening purposes and offers practical advantages in large-scale field studies, it may still introduce measurement error compared with laboratory-based venous blood assays. Finally, while the study was conducted in a representative sample from one region of Poland, the generalizability of the findings to other populations should be made with caution. Future multicenter or international studies are warranted to confirm the observed associations.

## 5. Conclusions

This study reveals that cardiometabolic risk factors are already present in childhood and adolescence.

Overweight and obesity affected over 18% of participants based on BMI and 26% based on body fat percentage, with abdominal obesity and stage I–II hypertension observed in nearly one-fourth of the sample. Children aged 6–12 years showed a higher prevalence of several metabolic abnormalities compared to adolescents.

Among adolescents, girls more frequently exhibited adverse lipid profiles, whereas boys had higher rates of hypertension. Elevated TG levels were independently associated with abdominal obesity and hypertension in girls, suggesting the need for early screening and preventive strategies tailored by sex and age.

Practical implications and future directions:

There is a clear need to implement early and widespread prevention and screening programs for metabolic disorders beginning in primary school age, including assessments of abdominal obesity and lipid profiles.

The findings indicate that TG concentration may serve as a simple and cost-effective screening marker for identifying girls at increased cardiometabolic risk.

There is also an urgent need for longitudinal studies to monitor the trajectory of metabolic changes over time and to better understand causal relationships.

## Figures and Tables

**Table 1:** Anthropometric, clinical, and biochemical characteristics of the study population by age group and sex.

Variables	Mean ± SD	Me (Q1 Q3)	Mean ± SD	Me (Q1 Q3)	Mean ± SD	Me (Q1 Q3)	*p*−Value
Children (6–12 years)	All n = 169 (100.0%)		Boys n = 75 (44.4%)		Girls n = 94 (55.6%)		
Age (years)	10.0 ± 1.50	10.0 (9.0–11.0)	10.0 ± 1.49	10.0 (9.0–11.0)	10.0 ± 1.52	10.0 (9.0–12.0)	Z = −0.09 0.929
Height (cm)	144.8 ± 11.69	144.5 (135.2–152.5)	145.5 ± 11.67	145.0 (136.0–151.0)	144.2 ± 11.74	143.8 (133.5–152.5)	Z = 0.57 0.571
Body weight (kg)	39.2 ± 12.35	37.4 (30.3–44.5)	40.3 ± 14.47	37.4 (30.1–45.2)	38.2 ± 10.34	37.9 (30.3–44.5)	Z = 0.35 0.729
BMI percentile	52.8 ± 28.94	52.0 (26.0–78.0)	52.0 ± 30.14	54.0 (23.0–77.0)	53.4 ± 28.10	51.0 (28.0–79.0)	Z = −0.27 0.790
Waist circumference (cm)	64.9 ± 8.64	64.0 (59.0–69.0)	65.8 ± 10.27	65.0 (58.0–68.0)	64.2 ± 7.06	64.0 (59.0–69.0)	Z = 0.35 0.723
Hip circumference (cm)	79.1 ± 9.38	78.0 (73.0–84.0)	79.3 ± 10.55	78.0 (72.0–83.0)	78.9 ± 8.38	78.5 (73.0–86.0)	Z = −0.34 0.735
Waist-to-hip ratio (WHR)	0.8 ± 0.06	0.8 (0.8–0.9)	0.8 ± 0.05	0.8 (0.8–0.9)	0.8 ± 0.06	0.8 (0.8–0.9)	t = 1.44 0.151
Systolic blood pressure (mmHg)	114.1 ± 12.22	113.5 (104.5–121.0)	115.2 ± 12.35	114.0 (106.0–124.0)	113.2 ± 12.10	112.3 (104.5–121.0)	t = 1.08 0.282
Diastolic blood pressure (mmHg)	67.6 ± 10.02	67.5 (61.0–74.0)	67.5 ± 9.99	68.5 (59.0–75.5)	67.6 ± 10.10	67.3 (62.0–73.0)	t = −0.07 0.947
Fasting glucose (mg/dL)	74.7 ± 9.32	74.0 (69.0–80.0)	75.5 ± 9.15	75.0 (70.0–81.0)	74.0 ± 9.44	73.5 (68.0–80.0)	t = 1.07 0.286
Total cholesterol (mg/dL)	149.6 ± 25.59	147.0 (133.0–165.0)	149.8 ± 27.09	147.0 (127.0–167.0)	149.4 ± 24.48	147.5 (133.0–165.0)	t = 0.11 0.916
HDL cholesterol (mg/dL)	51.2 ± 9.89	52.0 (44.0–58.0)	51.6 ± 10.19	52.0 (45.0–59.0)	51.0 ± 9.69	51.0 (44.0–57.0)	t = 0.40 0.688
LDL cholesterol (mg/dL)	98.3 ± 22.35	96.0 (83.0–113.0)	98.2 ± 23.59	94.0 (84.0–114.0)	98.4 ± 21.44	97.0 (82.0–109.0)	t = −0.06 0.955
TG (mg/dL)	88.5 ± 34.00	80.0 (70.0–94.0)	86.6 ± 31.61	79.0 (69.0–93.0)	90.1 ± 35.89	80.0 (70.0–95.0)	Z = −0.49 0.622
**Adolescents (13–17 years)**	**All** **n = 149 (100.0%)**		**Boys** **n = 70 (47.0%)**		**Girls** **n = 79 (53.0%)**		***p*-Value**
Age (years)	15.3 ± 1.48	15.0 (14.0–17.0)	15.4 ± 1.57	16.0 (14.0–17.0)	15.2 ± 1.40	15.0 (14.0–17.0)	Z = 0.64 0.524
Height (cm)	169.0 ± 8.77	168.0 (162.0–175.0)	174.8 ± 8.45	175.6 (169.0–181.5)	163.8 ± 5.03	163.0 (160.5–167.0)	t * = 9.50 <0.001
Body weight (kg)	60.5 ± 13.02	57.9 (51.2–67.9)	66.3 ± 14.34	66.2 (55.5–72.1)	55.4 ± 9.13	53.0 (49.0–60.5)	Z = 5.19 <0.001
BMI percentile	52.0 ± 28.76	55.0 (30.0–74.0)	55.2 ± 28.70	58.5 (31.0–79.0)	49.2 ± 28.70	50.0 (26.0–74.0)	Z = 1.35 0.177
Waist circumference (cm)	71.1 ± 9.54	69.0 (64.0–76.0)	74.9 ± 9.78	73.5 (68.0–79.0)	67.6 ± 7.89	66.0 (62.0–72.0)	Z = 5.01 <0.001
Hip circumference (cm)	92.9 ± 7.72	92.0 (88.0–98.0)	94.9 ± 8.24	94.5 (89.0–99.0)	91.2 ± 6.84	90.0 (87.0–95.0)	Z = 2.82 0.005
Waist-to-hip ratio (WHR)	0.8 ± 0.07	0.8 (0.7–0.8)	0.8 ± 0.05	0.8 (0.8–0.8)	0.7 ± 0.08	0.7 (0.7–0.8)	Z = 5.77 <0.001
Systolic blood pressure (mmHg)	117.3 ± 12.66	115.5 (109.0–127.5)	122.8 ± 13.58	122.3 (113.5–133.0)	112.3 ± 9.41	112.0 (105.0–117.0)	t * = 5.43 <0.001
Diastolic blood pressure (mmHg)	68.3 ± 7.72	68.5 (64.5–72.5)	68.1 ± 8.12	68.5 (63.5–73.0)	68.5 ± 7.39	68.5 (64.5–72.5)	t = −0.30 0.761
Fasting glucose (mg/dL)	75.5 ± 13.04	72.0 (67.0–84.0)	75.8 ± 13.14	73.5 (68.0–85.0)	75.2 ± 13.02	71.0 (66.0–84.0)	Z = 0.54 0.589
Total cholesterol (mg/dL)	140.3 ± 24.59	137.0 (122.0–151.0)	133.5 ± 18.65	134.0 (118.0–147.0)	146.3 ± 27.60	143.0 (126.0–159.0)	Z = −2.94 0.003
HDL cholesterol (mg/dL)	50.3 ± 12.14	48.0 (44.0–56.0)	46.6 ± 12.34	45.0 (39.0–53.0)	53.5 ± 11.07	52.0 (45.0–59.0)	Z = −3.75 <0.001
LDL cholesterol (mg/dL)	90.0 ± 22.39	88.0 (76.0–102.0)	86.9 ± 16.87	87.5 (76.0–97.0)	92.8 ± 26.13	89.0 (74.0–107.0)	Z = −0.95 0.340
TG (mg/dL)	93.3 ± 35.89	87.0 (70.0–101.0)	99.4 ± 41.22	87.0 (73.0–118.0)	87.8 ± 29.62	85.0 (68.0–100.0)	Z = 1.24 0.214

Bolded *p*-values indicate statistically significant differences between boys and girls; t—Student’s test; t *—Welch’s *t*-test; Z—Mann–Whitney U test.

**Table 2:** Prevalence of obesity and cardiometabolic risk factors according to age group and sex.

Parameters	All n (%)	Boys n (%)	Girls n (%)	*p*-Value
⁠	**Children (6–12 years)**			
Body weight status (CDC)				χ^2^ = 6.36 0.095
Underweight	5 (3.0)	3 (4.0)	2 (2.1)	
Normal	129 (76.3)	58 (77.3)	71 (75.5)	
Overweight	21 (12.4)	5 (6.7)	16 (17.0)	
Obesity	14 (8.3)	9 (6.7)	5 (5.3)	
Body fat status (McCarthy)				χ^2^ = 5.39 0.146
Underfat	1 (0.6)	1 (1.3)	0 (0.0)	
Normal	118 (69.8)	46 (61.3)	72 (76.6)	
Overfat	27 (16.0)	15 (20.0)	12 (12.8)	
Obesity	23 (13.6)	13 (17.3)	10 (10.6)	
Blood pressure category				χ^2^ = 3.04 0.219
Normal	87 (51.5)	54 (57.4)	33 (44.0)	
High normal	24 (14.2)	12 (12.8)	12 (16.0)	
Stage I and II hypertension	58 (34.3)	28 (29.8)	30 (40.0)	
Fasting glucose status				χ^2^ = 0.71 0.401
Normal	123 (72.8)	57 (76.0)	66 (70.2)	
Low/High	46 (27.2)	18 (24.0)	28 (29.8)	
Abdominal obesity				χ^2^ = 2.44 0.118
Yes	51 (30.2)	18 (24.0)	33 (35.1)	
No	118 (69.8)	57 (76.0)	61 (64.9)	
TG level				χ^2^ = 0.41 0.523
Acceptable	90 (53.3)	42 (56.0)	48 (51.1)	
Borderline/high	79 (46.7)	33 (44.0)	46 (48.9)	
HDL cholesterol level				χ^2^ = 0.37 0.542
Acceptable	113 (66.9)	52 (69.3)	61 (64.9)	
Borderline/low	56 (33.1)	23 (30.7)	33 (35.1)	
LDL cholesterol level				χ^2^ = 0.81 0.368
Acceptable	123 (72.8)	52 (69.3)	71 (75.5)	
Borderline/high	46 (27.2)	23 (30.7)	23 (24.5)	
Total cholesterol level				χ^2^ = 0.59 0.444
Acceptable	133 (78.7)	57 (76.0)	76 (80.9)	
Borderline/high	36 (21.3)	18 (24.0)	18 (19.1)	
	**Adolescents (13–17 years)**			
Body weight status (CDC)				χ^2^ = 1.22 0.748
Underweight	7 (4.7)	2 (2.9)	5 (6.3)	
Normal	118 (79.2)	56 (80.0)	62 (78.5)	
Overweight	13 (8.7)	7 (10.0)	6 (7.6)	
Obesity	11 (7.4)	5 (7.1)	6 (7.6)	
Body fat status (McCarthy)				χ^2^ = 1.48 0.687
Underfat	2 (1.3)	1 (1.4)	1 (1.3)	
Normal	114 (76.5)	55 (78.6)	59 (74.7)	
Overfat	20 (13.4)	7 (10.0)	13 (16.5)	
Obesity	13 (8.8)	7 (10.0)	6 (7.6)	
Blood pressure category				χ^2^ = 12.89 0.002 C = 0.13
Normal	106 (71.1)	40 (57.1)	66 (83.5)	
High normal	26 (17.5)	19 (27.1)	7 (8.9)	
Stage I and II hypertension	17 (11.4)	11 (15.7)	6 (7.6)	
Fasting glucose status				χ^2^ = 2.05 0.153
Normal	91 (61.1)	47 (67.1)	44 (55.7)	
Low/high	58 (38.9)	23 (32.9)	35 (44.3)	
Abdominal obesity				χ^2^ = 0.02 0.893
Yes	27 (18.1)	13 (18.6)	14 (17.7)	
No	122 (81.9)	57 (81.4)	65 (82.3)	
TG level				χ^2^ = 0.01 0.909
Acceptable	88 (59.1)	41 (58.6)	47 (59.5)	
Borderline/high	61 (40.9)	29 (41.4)	32 (40.5)	
HDL cholesterol level				χ^2^ = 9.71 0.002 C = 0.25
Acceptable	90 (60.4)	33 (47.1)	57 (72.2)	
Borderline/low	59 (39.6)	37 (52.9)	22 (27.8)	
LDL cholesterol level				χ^2^ = 7.85 0.005 C = 0.22
Acceptable	125 (83.9)	65 (92.9)	60 (75.9)	
Borderline/high	24 (16.1)	5 (7.1)	19 (24.1)	
Total cholesterol level				χ^2^ = 4.21 0.040 * C = 0.17
Acceptable	132 (88.6)	66 (94.3)	66 (83.5)	
Borderline/high	17 (11.4)	4 (5.7)	13 (16.5)	
	**Total sample**			
Body weight status (CDC)				χ^2^ = 2.65 0.449
Underweight	12 (3.8)	5 (3.4)	7 (4.0)	
Normal	247 (77.7)	114 (78.6)	133 (76.9)	
Overweight	34 (10.7)	12 (8.3)	22 (12.7)	
Obesity	25 (7.8)	14 (9.7)	11 (6.4)	
Body fat status (McCarthy)				χ^2^ = 2.40 0.493
Underfat	3 (0.9)	2 (1.4)	1 (0.6)	
Normal	232 (73.0)	101 (69.7)	131 (75.7)	
Overfat	47 (14.8)	22 (15.2)	25 (14.5)	
Obesity	36 (11.3)	20 (13.8)	16 (9.2)	
Blood pressure category				χ^2^ = 12.61 0.002 C = 0.20
Normal	193 (60.7)	73 (50.3)	120 (69.4)	
High normal	50 (15.7)	31 (21.4)	19 (11.0)	
Stage I and II hypertension	75 (23.6)	41 (28.3)	34 (19.7)	
Fasting glucose status				χ^2^ = 2.38 0.123
Normal	214 (67.3)	104 (71.7)	110 (63.6)	
Low/high	104 (32.7)	41 (28.3)	63 (36.4)	
Abdominal obesity				χ^2^ = 1.43 0.232
Yes	78 (24.5)	31 (21.4)	47 (27.2)	
No	240 (75.5)	114 (78.6)	126 (72.8)	
TG level				χ^2^ = 0.17 0.677
Acceptable	178 (56.0)	83 (57.2)	95 (54.9)	
Borderline/high	140 (44.0)	62 (42.8)	78 (45.1)	
HDL cholesterol level				χ^2^ = 3.14 0.076
Acceptable	203 (63.8)	85 (58.6)	118 (68.2)	
Borderline/low	115 (36.2)	60 (41.4)	55 (31.8)	
LDL cholesterol level				χ^2^ = 1.13 0.287
Acceptable	248 (78.0)	117 (80.7)	131 (75.7)	
Borderline/high	70 (22.0)	28 (19.3)	42 (24.3)	
Total cholesterol level				χ^2^ = 0.43 0.513
Acceptable	265 (83.3)	123 (84.8)	142 (82.1)	
Borderline/high	53 (16.7)	22 (15.2)	31 (17.9)	

Bolded *p*-values indicate statistically significant differences between boys and girls; Pearson’s chi-square test; * chi-square test with Yates’ correction for continuity; C—Pearson’s C contingency coefficient.

**Table 3:** Comparison of lipid profile by body mass status, abdominal obesity, and BP category in the total sample.

Variable	Total Cholesterol (mg/dL)	HDL Cholesterol (mg/dL)	LDL Cholesterol (mg/dL)	TG (mg/dL)
	Mean ± SD	Mean ± SD	Mean ± SD	Mean ± SD
Body weight status (CDC)				
Underweight and normal weight	145.0 ± 24.85	51.3 ± 11.11	93.7 ± 22.07	88.6 ± 32.28
Overweight and obesity	146.3 ± 28.43	48.4 ± 10.26	97.9 ± 25.27	100.3 ± 43.78
*p*	Z = 0.07 0.944	Z = 1.72 0.085	Z = −0.90 0.368	Z = −1.91 0.055
Body fat status (McCarthy)				
Underfat and normal body fat	146.0 ± 24.54	51.6 ± 11.45	94.4 ± 21.90	89.0 ± 32.48
Overfat and obesity	143.2 ± 28.15	48.4 ± 9.25	94.7 ± 25.03	95.6 ± 40.92
*p*	Z = 1.10 0.273	Z = 2.01 0.045	Z = 0.19 0.846	Z = −0.85 0.393
Abdominal obesity				
Yes	144.9 ± 24.62	48.9 ± 10.92	96.1 ± 20.89	101.9 ± 46.07
No	145.3 ± 25.84	51.4 ± 10.97	93.9 ± 23.30	87.1 ± 29.67
*p*	Z = −0.18 0.859	Z = 1.88 0.061	Z = −0.82 0.415	**Z = −2.25** **0.025**
Blood pressure category				
Normal	144.4 ± 26.07	50.8 ± 10.56	93.6 ± 23.83	88.8 ± 34.07
High normal	148.3 ± 28.05	51.3 ± 13.64	97.0 ± 21.58	93.3 ± 34.76
Stage I and II hypertension	145.3 ± 22.25	50.4 ± 10.27	94.9 ± 20.54	93.9 ± 37.29
*p*	H = 0.98 0.613	H = 0.35 0.841	H = 1.37 0.505	H = 2.02 0.363

Bolded *p*-values indicate statistically significant differences; Z—Mann–Whitney U test; H—Kruskal–Wallis test.

**Table 4:** Association between lipid profile abnormalities and obesity, elevated BP, and glucose levels in girls (univariate analysis).

	Girls				Boys			
Variable	Acceptable	Borderline/High		*p*	Acceptable	Borderline/High		*p*
	n (%)	n (%)	OR (95% p.u.)		n (%)	n (%)	OR (95% p.u.)	
	TG level				TG level			
Abdominal obesity	19 (21.1)	28 (35.9)	2.09 (1.05–4.15)	0.033	17 (20.5)	14 (22.6)	1.13 (0.51–2.52)	0.760
Abnormal glucose level	30 (31.6)	33 (42.3)	1.54 (0.82–2.90)	0.177	27 (32.5)	14 (22.6)	0.60 (0.29–1.28)	0.188
Stage I–II hypertension	13 (14.4)	21 (26.9)	2.18 (1.01–4.72)	0.045	27 (32.5)	14 (22.6)	0.60 (0.29–1.28)	0.188
	LDL cholesterol level				LDL cholesterol level			
Abdominal obesity	38 (29.0)	9 (24.3)	0.79 (0.34–1.82)	0.575	26 (22.2)	5 (17.9)	0.76 (0.26–2.20)	0.613
Abnormal glucose level	48 (36.6)	15 (35.7)	1.05 (0.50–2.24)	0.894	32 (27.4)	9 (32.1)	1.26 (0.52–3.07)	0.613
Stage I–II hypertension	28 (21.4)	6 (16.2)	0.71 (0.27–1.88)	0.491	33 (28.2)	8 (28.6)	1.02 (0.41–2.54)	0.969
	HDL cholesterol level				HDL cholesterol level			
Abdominal obesity	30 (25.4)	17 (34.0)	1.51 (0.74–3.10)	0.258	16 (18.8)	15 (25.0)	1.44 (0.65–3.19)	0.372
Abnormal glucose level	45 (38.1)	18 (32.7)	0.84 (0.42–1.67)	0.612	28 (32.9)	13 (21.7)	0.56 (0.26–1.21)	0.138
Stage I–II hypertension	23 (19.5)	11 (22.0)	1.16 (0.52–2.62)	0.711	27 (31.8)	14 (23.3)	0.65 (0.31–1.39)	0.267

**Table 5:** Multivariate logistic regression analysis of the association between lipid profile abnormalities and health outcomes in girls and boys.

Variable	Girls		Boys	
	OR (95% p.u.)	*p*	OR (95% p.u.)	*p*
	Abdominal obesity			
TG level	2.36 (1.18–4.72)	0.015	1.07 (0.47–2.42)	0.868
LDL cholesterol level	0.58 (0.25–1.37)	0.214	0.78 (0.27–2.25)	0.642
HDL cholesterol level	1.31 (0.64–2.70)	0.463	1.41 (0.62–3.17)	0.413
	Abnormal glucose level			
TG level	1.62 (0.86–3.03)	0.134	0.65 (0.30–1.40)	0.272
LDL cholesterol level	0.91 (0.44–1.89)	0.797	1.25 (0.51–3.09)	0.627
HDL cholesterol level	0.78 (0.39–1.53)	0.465	0.61 (0.28–1.33)	0.213
	Stage I–II hypertension			
TG level	2.47 (1.13–5.38)	**0.023**	0.64 (0.30–1.38)	0.255
LDL cholesterol level	0.54 (0.20–1.43)	0.215	1.02 (0.40–2.56)	0.971
HDL cholesterol level	1.02 (0.45–2.32)	0.956	0.71 (0.33–1.52)	0.373

## Data Availability

Due to personal data protection reasons, all data are available from the authors of this publication.

## Abbreviations

The following abbreviations are used in this manuscript:

LDL	Low-Density Lipoprotein
HDL	High-Density Lipoprotein
OR	odds ratio
BMI	body mass index
BIA	bioelectrical impedance analysis
WHO	World Health Organization
HC	hip circumference
BP	blood pressure
SBP	systolic blood pressure
DBP	diastolic blood pressure
ISAK	International Society for the Advancement of Kinanthropometry
WHR	waist-to-hip ratio
WC	Waist circumference
CDC	Centers for Disease Control and Prevention
COSI	Childhood Obesity Surveillance Initiative
WHtR	Waist-to-Height Ratio
CI	confidence intervals
